# Supplementary material for: Lower Extremity Arterial Disease and Lumbar Spinal Stenosis: A Study of Exercise-Induced Arterial Ischemia in 5197 Patients Complaining of Claudication
Source: J Clin Med. 2022 Sep 22;11(19):5550. doi: 10.3390/jcm11195550 (PMC9572820; doi:10.3390/jcm11195550)
Supplement: Supplementary file 1 [file jcm-11-05550-s001.zip › jcm-1894046-supplementary.pdf]

File S1: Sub group analysis

Lower extremity arterial disease and lumbar spinal stenosis: sub-study of exercise-induced arterial proximal ischemia in 2416 patients complaining proximal claudication.

1. Introduction

In could be suggested that vascular and neurogenic claudication are specifically of interest in case of proximal symptoms, because Ischemia has been proposed as a cause of lumbar disease, and because symptoms of proximal claudication can mimic neurogenic claudication [1-3]. It has been suggested that muscle ischemia is not the origin of symptoms in most patients with neurogenic claudication [4]. This population is also of particular interest because ankle-brachial index (ABI) at rest is frequently normal if lesions are not located on the measured axis (such as lesions of the internal iliac artery) [5-7]. Further transcutaneous oximetry is of particular interest at the proximal (Buttock) level [7, 8]. Then we repeated the study in a sub-group of patients reporting pain by history or on treadmill at the buttock level.

2. Materials and Methods

Population

From the initial population reported in the manuscript, we selected the patients that reported buttock exercise-induced pain either by history or on treadmill.

Exercise-oximetry recordings

For this specific group we considered exercise oximetry results as being positive only in the presence of unilateral or bilateral buttock ischemia (buttock DROPmin< –15 mmHg).

Score determination and validation

Age and BMI were dichotomized from their respective median values and male/female sex was encoded as 1/0 as for the whole population. Thereafter, score determination and validation were performed as in the principal study.

Statistical analysis.

Results and tests were performed as in the main manuscript.

3. Results

When focusing on patients reporting buttock claudication by history or on treadmill when had 2416 patients (figure 1′). Of them, 351 (14.5 %) patients had a history of LSS which is slightly higher than the one observed for the whole population.

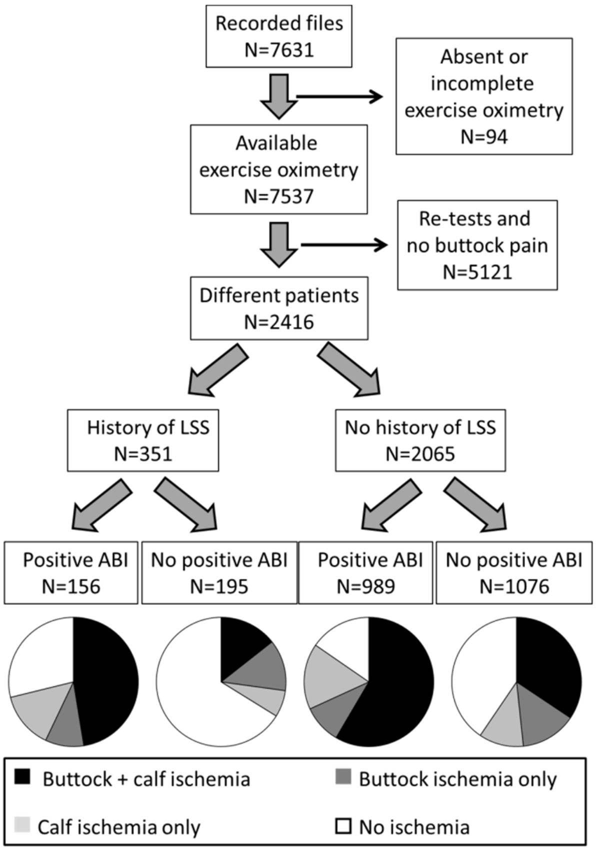

Figure 1'. Flowchart of the study and distribution of the presence/absence of a history of suspected, diagnosed, or treated lumbar spinal stenosis (LSS), an abnormal (positive) ankle-brachial index (ABI) defined as ABI < 0.90 or > 1.40. The presence and localization of exercise-induced ischemia on exercise-oximetry (TcpO2) are reported for each group of patients using circle graphs.

Characteristics of patients in this specific sub-study are reported in table 1'.

Table 1'. Characteristics of the sub-group of patients complaining proximal claudication. LEAD stands for lower extremity artery disease, and ABI stands for ankle-brachial index.

	History of LSS n=351	No history of LSS n=2065	p
Male sex	284 (80.9)	1681 (81.4)	0.824
Weight (kg)	81.5 ± 15.2	76.9 ± 15.4	0.001
Height (cm)	169 ± 9	169 ± 8	0.768
Age (years old)	67.4 ± 9.7	62.8 ± 11.3	0.001
Antiplatelet agent	223 (63.5)	1485 (71.9)	0.002
Antihypertensive drugs	220 (62.7)	1166 (56.5)	0.031
Cholesterol lowering drugs	201 (57.3)	1187 (57.5)	0.953
Diabetes mellitus	381 (18.5)	88 (25.1)	0.005
Active smokers	53 (15.0)	317 (15.4)	0.936
Pain by history on left buttock	281 (80.1)	1300 (63.0)	0.001
Pain by history on right buttock	281 (80.1)	1318 (63.8)	0.001
Pain by history on left thigh	97 (27.6)	469 (22.7)	0.048
Pain by history on right thigh	95 (27.1)	474 (23.0)	0.102
Pain by history on left calf	146 (41.6)	852 (41.3)	0.907
Pain by history on right calf	147 (41.9)	881 (42.7)	0.815
Time over 10 m (sec)	10.4 ± 2.7	9.8 ± 2.3	0.001
History of cardiovascular disease	171 (48.7)	1394 (67.5)	0.001
History of lower limb revascularization	53 (15.1)	363 (17.6)	0.284
LEAD according to ABI (<0.90 or >1.40)	156 (44.4)	989 (47.9)	0.248
Right positive ABI	121 (34.5)	776 (37.6)	0.282
Left positive ABI	113 (32.2)	839 (40.6)	0.003

As shown in table 1′, patients were predominantly males and patients with a history of LSS were older and heavier than the patients without a history of LSS. Note that, many patients had a history of cardiovascular disease but direct access to lower limb vascular imaging was rarely available at referral (<10% of cases).

Results of the treadmill tests observed in the population are reported in table 2′. Of interest is to note that the patients with a history of LSS reported buttock pain more frequently by history than the other patients while the prevalence of buttock pain on treadmill more similar in the two groups. As also reported in table 2′, no difference was found on walking time on treadmill shorter walking time, and due to a higher prevalence of positive tests both at the buttock and calf levels, the median value of DROPm was lower in patients without a history of LSS that in patients with a history of LSS.

Table 2′. Results of the treadmill test with exercise-oximetry in patients complaining proximal claudication with or without a history of suspected diagnosed or treated, lumbar spinal stenosis. Exercise-oximetry results are expressed in absolute values (TcpO2), and in lowest decrease from rest of oxygen (DROPmin) index.

	History of LSS n=351	No history of LSS n=2065	p
Maximal walking time (sec)	288 [161;563]	275 [173;498]	0.808
Heart rate at rest (beats/min)	79 ± 15	80 ± 15	0.173
Heart rate at end exercise (beats/min)	117 ± 21	119 ± 22	0.152
Chest TcpO2 at rest (mm Hg)	67 ± 12	67 ± 14	0.781
Minimal chest TcpO2 (mm Hg)	62 ± 12	62 ± 14	0.496
Left buttock TcpO2 at rest (mm Hg)	68 ± 11	69 ± 13	0.210
Left calf TcpO2 at rest (mm Hg)	70 ± 10	71 ± 12	0.010
Right buttock TcpO2 at rest (mm Hg)	68 ± 10	69 ± 13	0.156
Right calf TcpO2 at rest (mm Hg)	70 ±10	71 ± 12	0.078
Left buttock DROPmin (mm Hg)	-9 [-18;-5]	-14 [-25;-7]	0.001
Left calf DROPmin (mm Hg)	-10 [-18;-6]	-14 [-25;-7]	0.001
Right buttock DROPmin (mm Hg)	-9 [-18;-5]	-14 [-25;-7]	0.001
Right calf DROPmin (mm Hg)	-9 [-17;-5]	-14 [-25;-8]	0.001
DROPmin<-15 mmHg on one or both buttocks	142 (40.5)	1194(57.8)	0.001
DROPmin<-15 mmHg on one or both calves	137 (39.06)	1231 (59.6)	0.001
Left buttock pain on treadmill	214/351 (61.0)	1199 (58.1)	0.320
Right buttock pain on treadmill	206 (58.7)	1208 (58.5)	0.953
Left thigh pain on treadmill	33 (9.4)	143 (6.9)	0.119
Right thigh pain on treadmill	32 (9.1)	154 (7.5)	0.279
Left calf pain on treadmill	149 (42.5)	945 (45.8)	0.271
Right calf pain on treadmill	142 (40.5)	953 (46.2)	0.049

As shown in figure 1 and as expected, most patients with an abnormal ABI had a significant ischemia during the treadmill test. Specifically, the proportions of results showing exercise-induced proximal ischemia (regardless of the presence or absence of associated distal ischemia) were respectively 57.1 % in patients with a history of LSS and ABI+, 27.2 % in patients with a history of LSS and ABI-, 68.1 % in patients without a history of LSS and ABI+, and still 48.3 % in patients without a history of LSS and ABI- patients. These patients are presented in black and dark grey in the figures. Note that in all cases, an additional amount of patients showed calf ischemia only. Interestingly, more than half of the patients without a history of LSS with positive arterial results showed ischemia at the proximal level. Inversely, 15.4 % of these patients without a history of LSS showed no ischemia at exercise despite the presence of an abnormal ABI. This suggests that the limiting factor for exercise in these patients was not ischemia, among which possibly unknown LSS, non-neurogenic causes such as osteo-articular or cardio-respiratory diseases. Also notable is that, even with a normal ABI at rest, one third of the patients with a history of LSS showed a significant ischemia on treadmill, suggesting that a vascular origin was responsible of (or at least participated to) the limitation of walking capacity.

For the regression analysis of the factors associated with the presence of exercise-induced ischemia in the patients with a history of LSS (table 3′), 6 significant factors were identified (r=0.378; p<0.001). These factors were male sex, ABI+, antiplatelet treatment, BMI < 26.5 kg/m², a history of lower limb revascularization, and age ≤ 63 years old.

Table 3′. Results from the step-by-step linear regression analysis in patients with buttock pain by history or on treadmill and without lumbar spinal stenosis of factors predictive of the presence of proximal exercise-induced ischemia. The points for the score are the alpha non decimal values obtained as explained in the text

Studied parameters	Beta	SE	Normalised beta	p	Points for the score
Male sex	0.292	0.026	0.230	<0.001	+3
ABI+	0.161	0.021	0.163	<0.001	+2
Antiplatelet treatment	0.139	0.023	0.126	<0.001	+2
BMI < 26.5 kg/m²	0.069	0.021	0.070	<0.001	+1
Age ≤ 63 years old	0.069	0.021	0.070	0.001	+1
Lower limb revascularization	0.082	0.027	0.064	0.023	+1
Antihypertensive drugs	-0.006	0.267		0.790	-
Cholesterol lowering treatment	0.010	0.430		0.667	-
Diabetes mellitus	-0.032	1.496		0.135	-
Active smoking	0.015	0.735		0.016	-

The smallest κ coefficient to be used was 9 for buttock ischemia only, and the one resulting in the smallest deviation of rounded score from the non-rounded score was 14. After multiplying all beta values in the regression model by 14 and rounding to the closer unit, the α values in the prediction model were 3, 2, 2, 1, 1 and 1 respectively, resulting in a score ranging from 0 to 10 points. This score showed an area under ROC curve of 0.691 ± 0.012; p<0.001 for patients without a history of LSS.

When the score defined in patients without a history of LSS was applied to the patients with a history of LSS, the probability of the presence of exercise induced ischemia was proportional to the score (Figure 2′).

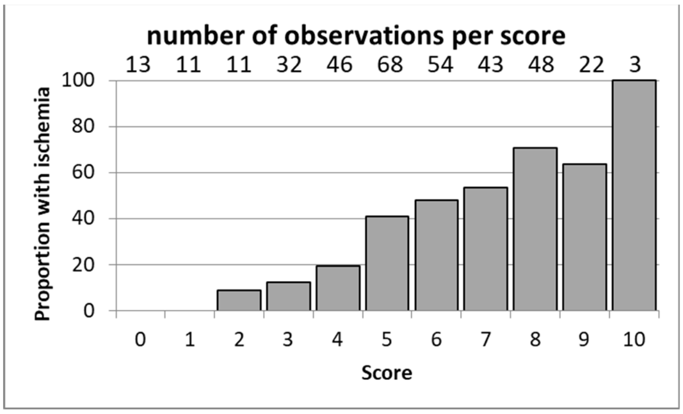

Figure 2′: Proportion of patients with a history of lumbar spinal stenosis showing proximal only exercise-induced ischemia as a function of the score defined in table 3′.

For buttock ischemia, the proportion of patients with exercise-induced ischemia was equal or lower than 20% for scores < 5 (which implies having ABI-), while it exceeded 60% for scores >7 (which implies being a male plus either having antiplatelet treatment or ABI+, plus having at least one of the other three factors or being a male plus having both antiplatelet treatment and ABI+ plus having at least one of the other three factors).

The area under ROC curve in patients with a history of LSS was 0.755±0.056; p<0.001 with 142 cases with and 209 cases without buttock ischemia (Figure 3′).

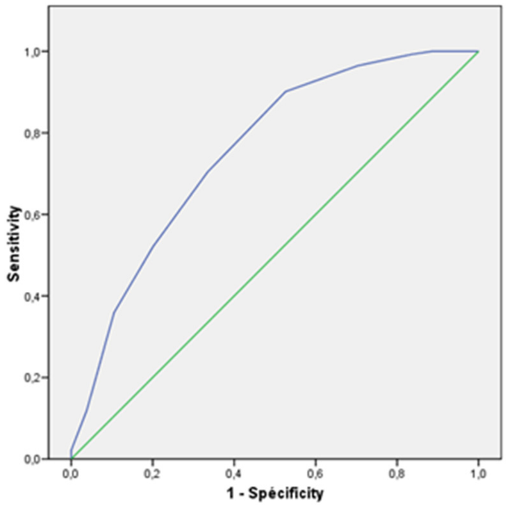

Figure 3':_Receiver operating characteristics curves for the score to predict the presence of proximal or distal (left panel) or proximal only (right panel) exercise-induced ischemia in patients with a history of lumbar spinal stenosis.

4. Discussion

The focus on the sub-group of patients complaining proximal (Buttock) claudication is of interest because these patients represent a group of specific diagnostic difficulty. Note that it is important to account for both the symptoms reported by history and the symptoms observed on treadmill, since it is frequently observed that patients complaining calf claudication do not spontaneously report their proximal symptoms [9]. The present sub-study provides results that are very close to the results observed in the general population with the same six factors (Including dichotomization on Median age and BMI). As illustrated in figure 1', although almost 1/3 of the patients with a normal ABI in the LSS group have exercise induced ischemia, ABI remains an important –although imperfect- factor in the prediction of positive results and doubles the risk of positive exercise oximetry.

5. References

1.Murphy M.A., Denton M.J., Scott D.F. Neurogenic claudication secondary to vascular disease. *Aust N Z J Surg*. **1992**;*62*:154-7.
2.Wohlgemuth W.A., Stoehr M. Percutaneous arterial interventional treatment of exercise-induced neurogenic intermittent claudication due to ischaemia of the lumbosacral plexus. *J Neurol*. **2002**;*249*:988-92.
3.Wohlgemuth W.A., Rottach K.G., Stoehr M. Intermittent claudication due to ischaemia of the lumbosacral plexus. *J Neurol Neurosurg Psychiatry*. **1999**;*67*:793-5.
4.Keenan G.F., Ashcroft G.P., Roditi G.H., Hutchison J.D., Evans N.T., Mikecz P., et al. Measurement of lower limb blood flow in patients with neurogenic claudication using positron emission tomography. *Spine (Phila Pa 1976)*. **1995**;*20*:408-11.
5.Gernigon M., Marchand J., Ouedraogo N., Leftheriotis G., Piquet J.M., Abraham P. Proximal ischemia is a frequent cause of exercise-induced pain in patients with a normal ankle to brachial index at rest. *Pain Physician*. **2013**;*16*:57-64.

6. Abraham P., Hersant J., Ramondou P., Picquet J., Feuilloy M., Henni S., et al. Comparison of exercise oximetry and ankle pressure measurements for patients with intermittent claudication: an observational study of 433 patients. *Pflugers Arch*. **2020**;*472*:293-301.
7. Abraham P., Ramondou P., Hersant J., Sempore W.Y., Feuilloy M., Henni S. Investigation of arterial claudication with transcutaneous oxygen pressure at exercise: Interests and limits. *Trends Cardiovasc Med*. **2021**;*31*:218-23.
8. Abraham P., Picquet J., Vielle B., Sigaudo-Roussel D., Paisant-Thouveny F., Enon B., et al. Transcutaneous oxygen pressure measurements on the buttocks during exercise to detect proximal arterial ischemia: comparison with arteriography. *Circulation*. **2003**;*107*:1896-900.
9. Jaquinandi V., Picquet J., Bouye P., Saumet J.L., Leftheriotis G., Abraham P. High prevalence of proximal claudication among patients with patent aortobifemoral bypasses. *J Vasc Surg*. **2007**;*45*:312-8.
